# Label-free neuroblastoma cell separation from hematopoietic progenitor cell products using acoustophoresis - towards cell processing of complex biological samples

**DOI:** 10.1038/s41598-019-45182-3

**Published:** 2019-06-19

**Authors:** Franziska Olm, Anke Urbansky, Josefina H. Dykes, Thomas Laurell, Stefan Scheding

**Affiliations:** 1 Lund Stem Cell Center & Division of Molecular Haematology, Department of Laboratory Medicine, Lund University, 221 00 Lund, Sweden; 2 Division of Nanobiotechnology and Lab-on-a-chip, Department of Biomedical Engineering, Lund University, 221 00 Lund, Sweden; 3 Department of Haematology, Skåne University Hospital, 222 41 Lund, Sweden; 4 Division of Haematology and Transfusion Medicine, Department of Laboratory Medicine, University and Regional Laboratories, 221 84 Lund, Sweden

**Keywords:** Translational research, Preclinical research

## Abstract

Processing of complex cell preparations such as blood and peripheral blood progenitor cell (PBPC) transplants using label-free technologies is challenging. Transplant-contaminating neuroblastoma cells (NBCs) can contribute to relapse, and we therefore aimed to provide proof-of-principle evidence that label-free acoustophoretic separation can be applied for diagnostic NBC enrichment and removal (“purging”) from human blood and PBPC products. Neuroblastoma cells spiked into blood and PBPC preparations served as model systems. Acoustophoresis enabled to enrich NBCs from mononuclear peripheral blood cells and PBPC samples with recovery rates of up to 60–97%. When aiming at high purity, NBC purities of up to 90% were realized, however, compromising recovery. Acoustophoretic purging of PBPC products allowed substantial tumour cell depletion of 1.5–2.3 log. PBPC loss under these conditions was considerable (>43%) but could be decreased to less than 10% while still achieving NBC depletion rates of 60–80%. Proliferation of cells was not affected by acoustic separation. These results provide first evidence that NBCs can be acoustically separated from blood and stem cell preparations with high recovery and purity, thus indicating that acoustophoresis is a promising technology for the development of future label-free, non-contact cell processing of complex cell products.

## Introduction

Complex biological samples such as blood and stem cell products are routinely processed to prepare cells for subsequent analytical or therapeutic purposes. Currently, centrifugation is often the standard initial step in such procedures. However, it usually needs to be combined with more targeted cell separation approaches when aiming for the isolation of specific cellular components.

Microfluidic-based acoustophoresis, which utilizes ultrasonic standing wave forces to control particle movement, has emerged as a possible alternative cell separation method^1–8^. Acoustophoretic separation is primarily based on size, density, and compressibility of the particles in relation to the suspending medium (see equations 1 and 2, Fig. S1 and the video animations for illustration of the separation principle in the supplementary information). This allows to more selectively separate specific cell types without the use of antibody labelling technology, provided that the acoustic properties of the target cells are sufficiently different from the non-target cell population. The suitability of acoustophoresis for cell separation has been demonstrated for a number of different areas including human cell products^4,7,9–11^. Acoustophoresis demonstrated potential to provide simple, cost-effective, and gentle cell handling, while having no impact on cell function and survival^11–14^.

Based on our previous clinically-directed applications^1,5,6^, this study aimed to establish the label-free separation of neuroblastoma cells from blood and peripheral blood progenitor cell (PBPC) products. Neuroblastoma (NB) is an early childhood cancer with poor survival rates in high-risk patients. The treatment with intensive chemotherapy and autologous stem cell transplantation has improved the outcome for these patients but nevertheless, disease relapse is still a major problem and survival rates are only about 40–50%^15–17^. Circulating tumour cells (CTCs) and stem cell product-contaminating NBCs, which can be detected in the blood of about 70% of high-risk neuroblastoma patients and 50% of stem cell collections^15,18–20^, respectively, carry important diagnostic and prognostic information, which motivates the development of efficient tumour cell isolation methods.

Furthermore, stem cell graft-contaminating tumour cells have been demonstrated to contribute to relapse after autologous bone marrow transplantation^21^, which provides the rationale to develop strategies to remove tumour cells (“purging”) from the graft to decrease relapse risk^16,22–24^. However, there is an ongoing controversial debate about a possible clinical advantage of tumour cell graft purging in neuroblastoma^25^. Handgretinger *et al*. for example provided surprising evidence for positive effects of reinfused tumour cells on survival rates^26^. But nevertheless, transplantation of a tumour cell depleted or even tumour cell free stem cell graft seems preferable to avoid retransfusion of viable tumour cells.

**Figure 1:**
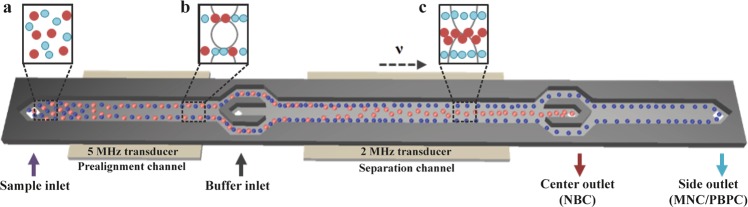
Schematic drawing of the acoustophoresis chip (total length 62.6 mm). (**a**) The MNC and PBPC suspension (represented by blue dots) spiked with neuroblastoma cells (red dots) is aspirated into the chip through the sample inlet at 100 μL/min. (**b**) In the prealignment channel (resonator operated at 5 MHz) cells are lined up in two parallel bands. Sorting buffer is infused with 300 μL/min through the buffer inlet to improve separation resolution. (**c**) The aligned cells enter the separation channel (resonator operated at 2 MHz), where the second acoustic field affects their lateral positioning depending on the acoustic properties of the cells (see equation 1, supplementary information). Tumour cells experience higher radiation forces than the smaller blood cells and thus are moved to the center of the channel and collected in the center outlet. MNCs/PBPCs remain close to the channel walls and exit through the side outlet.

In this paper, we approached to develop acoustophoresis as a potential label-free tool for neuroblastoma cell enrichment and PBPC graft purging in a model system using NB cell line-spiked blood and PBPC samples. The data showed sufficient acoustophysical differences between blood cells, PBPCs and NBCs, and by optimizing experimental conditions we provide first proof-of-principle evidence for efficient isolation of viable neuroblastoma cells from blood mononuclear cells (MNCs) and PBPC products using our standard acoustophoresis chip (Fig. 1).

## Results

### Blood cells and neuroblastoma cells differ in size and acoustic properties

**Figure 2:**
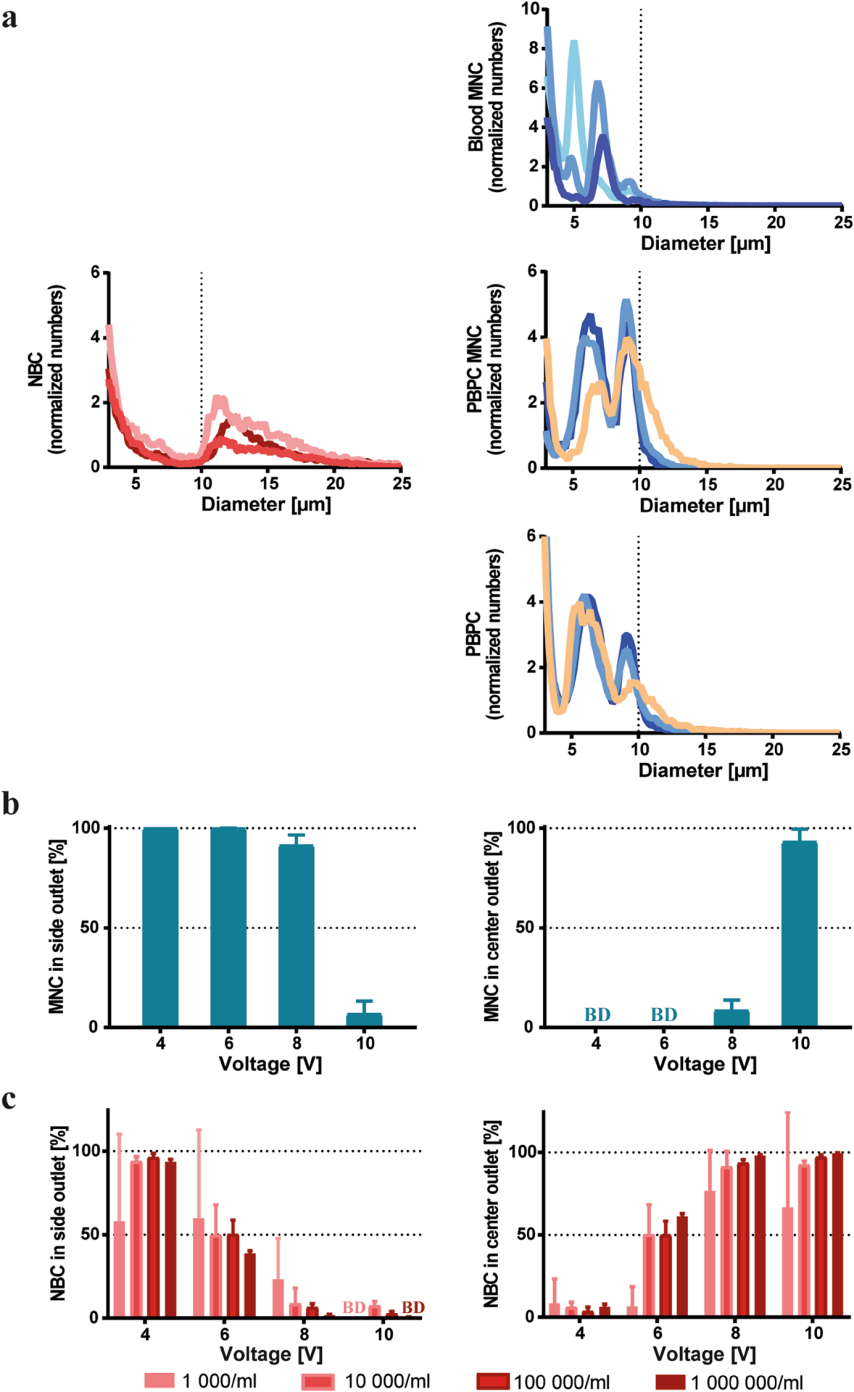
Blood cells, PBPCs and neuroblastoma cells differ in size and acoustic properties. (**a**) Cell size distributions of different peripheral blood cell preparations and neuroblastoma cells. SH-SY5Y neuroblastoma cells (left), blood MNCs (upper right), PBPC MNCs (middle right), and unprocessed PBPCs (lower right) are shown. PBPC MNC and PBPC samples were from the same donors. The different PBPC donors are indicated by orange: healthy donor, blue: myeloma patients. Different shades of the same colour present different passages (red) or different donors, respectively (blue). Cell numbers are shown as normalized cell counts relative to the total number of cells acquired for each individual sample (n = 3). Counts in the small size range account for dead or apoptotic cells as well as debris in the samples. (**b**,**c**) Peripheral blood MNCs and NB cells behave differently in the acoustophoresis chip. MNCs (1 × 10^6^ cells/mL) and NBCs (1 × 10^3^–1 × 10^6^ cells/mL) were evaluated separately. (**b**) MNCs collected in the side outlet (left) and center outlet (right) when exposed to different acoustic field strengths by increasing voltages on the separation channel. (**c**) NBCs collected in the side outlet (left) and center outlet (right) using the same voltages as in (**b**). The fraction of cells collected per outlet is expressed as percent of total collected cells. Data are shown as mean ± SD of 3 independent experiments. BD represents data below the detection limit.

The efficiency in separating different cell types with acoustophoresis is depending on differences in their acoustophysical properties. Here, size is an important parameter, as the acoustic radiation force scales with the radius to the third power (see equation 1, supplementary materials). Therefore, we first analysed the cell diameter distributions of peripheral blood and PBPC preparations, and the neuroblastoma cell line SH-SY5Y (Fig. 2).

Tumour cells ranged mostly from 10–20 µm and showed a stable size distribution independent of passage number (Fig. 2a, left). Smaller sized tumour cells were identified as dead or apoptotic by flow cytometry and aggregated cells accounted for larger diameter counts (data not shown). Blood MNCs (Fig. 2a, upper right) showed a main size peak between 6–8 µm. The majority of cell diameters for processed and unprocessed PBPC samples ranged between 5–12 µm. The composition of stem cell preparations also differed between healthy donors (Fig. 2a, middle and lower right, orange line; Fig. S2) and patients, as well as between different patient PBPCs (Fig. 2a, middle and lower right, blue lines; Fig. S2), which resulted in variations in size distributions of the cell populations. Consequently, the size overlap between different stem cell preparations and tumour cells varied somewhat from donor to donor. Nevertheless, these data provide evidence that blood, as well as PBPC preparations, and the tested neuroblastoma cells have different cell size distributions with a minor size overlap.

We therefore proceeded to test whether or not these differences would translate into differential acoustic mobilities of peripheral blood MNCs and NBCs in the acoustic field. As shown in Fig. 2b, more than 90% of the MNCs were sorted into the side outlet for voltages up to 8 V and were only directed into the center outlet at higher voltages. In contrast, neuroblastoma cells were effectively moved into the center outlet at voltages as low as 6 V (Fig. 2c). The observed high standard deviations for some data points when running samples at low cell concentrations resulted from the low numbers of cells sorted into a certain outlet which were close to the detection limit of the flow cytometer. In some experiments, no cells were detected in a certain outlet and, therefore, the sums of both outlets did not reach 100% for all data points. Nevertheless, these experiments, in which the different cell types were evaluated separately, thus confirmed that MNCs and NBCs differed in acoustic properties, which is the prerequisite for the sorting of contaminating tumour cells from blood or stem cell preparations.

### Tumour cell enrichment from peripheral blood samples for diagnostic purposes

Tumour cell enrichment for diagnostic purposes aims at the isolation of a sufficient number of tumour cells from a given cell source with adequate purity. Therefore, and in contrast to tumour cell purging, high recovery of non-tumour cells is not a priority.

**Figure 3:**
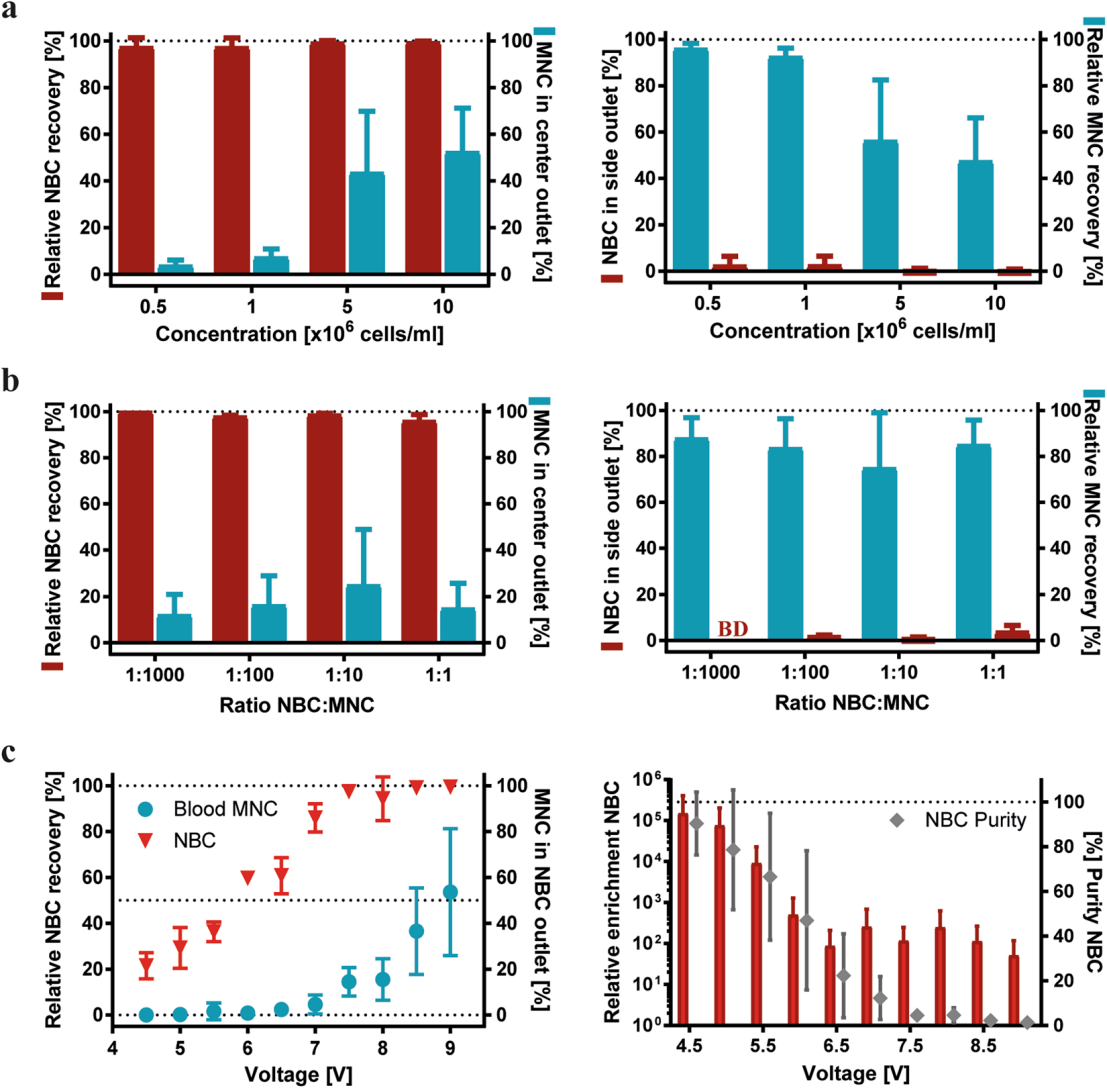
Acoustic separation of blood MNCs (blue) and NBCs (red). (**a**) Impact of sample concentration (0.5 × 10^6^–1 × 10^7^ cells/mL, spiking ration 1:1), and (**b**) spiking ratio (1:1–1:1,000, 1 × 10^6^ cells/mL) on separation outcome in center (left) and side (right) outlets in 3 independent experiments. BD represents data below the detection limit. (**c** left) Relative NBC recovery and MNC collected in the center outlet [%], when separating spiked NBC from MNC with an initial ratio of one NBC in 100 MNCs, an initial sample concentration of 1 × 10^6^ cells/mL, and a sample throughput of 100 µl/min (100,000 cells/min, n = 3–7). (**c** right) Relative enrichment of NBCs to MNCs compared to input samples, and purity [%] (diamonds) of enriched NBCs in the center outlet after separation. All values are given as mean ± SD for viable cells.

We investigated if NBCs could be effectively isolated for diagnostic purposes, using tumour cell-spiked blood MNC preparations as a model system (Fig. 3). The data showed that NBCs were recovered with over 97% in the center outlet independent of the sample concentration, but MNC recovery in the side outlet decreased by almost 40% at concentrations of 5 × 10^6^ cells/mL and higher for equally spiked input samples (Fig. 3a). Independent of the spiking ratios, NBC recoveries in the center outlet were as high as 96–100%, as shown in Fig. 3b, whereas NBC levels in the side outlet were very low or below the detection limit, respectively.

Next, the effects of increasing acoustic forces on NBC enrichment were investigated using a fixed cell concentration of 1 × 10^6^ cells/mL and a spiking ratio of 1:100 (Fig. 3c). Representative FACS plots are shown in Supplementary Fig. S3. The relative tumour cell recovery in the center outlet increased steadily with increasing forces from 21.46 ± 5.72% at 4.5 V to 85.97 ± 6.18% at 7 V, while only low numbers of MNCs between 0.03 ± 0.04% and 4.59 ± 4.13% contaminated the center outlet fraction. For higher voltages, virtually all NBCs (99.46 ± 0.56%) were collected in the center outlet, however, the number of contaminating MNCs increased considerably to up to 53.61 ± 27.65% (Fig. 3c, left). NBC purities of up to 90.32 ± 14.0% were achieved at low voltages, decreasing to only 1.33 ± 0.35% at the highest voltage tested. These low purities were caused by the increasing numbers of contaminating MNCs in the center outlet at higher acoustic forces. Consequently, relative enrichment rates of NBCs decreased as well (Fig. 3c, right).

Taken together, optimal conditions for the enrichment of viable tumour cells from blood MNCs for diagnostic purposes could be achieved when using lower acoustic forces (here generated with 4.5 and 5 V), which allowed to obtain highly purified tumour cell populations (>80%).

### Tumour cell enrichment and purging from peripheral blood progenitor cell preparations

Tumour cells isolated from PBPC products from patients with high-risk neuroblastoma undergoing autologous PBPC transplantation can provide valuable diagnostic information. As mentioned before, high tumour cell recovery and purity are targeted when aiming for tumour cell enrichment. Purging, on the other hand, aims for a complete removal of tumour cells from the autologous cell graft, while maximally recovering cells relevant for transplantation, i.e. the hematopoietic stem and progenitor cells. Other cell types present in the graft such as lymphocytes and platelets are not essential for engraftment and, thus, a certain loss is acceptable.

**Figure 4:**
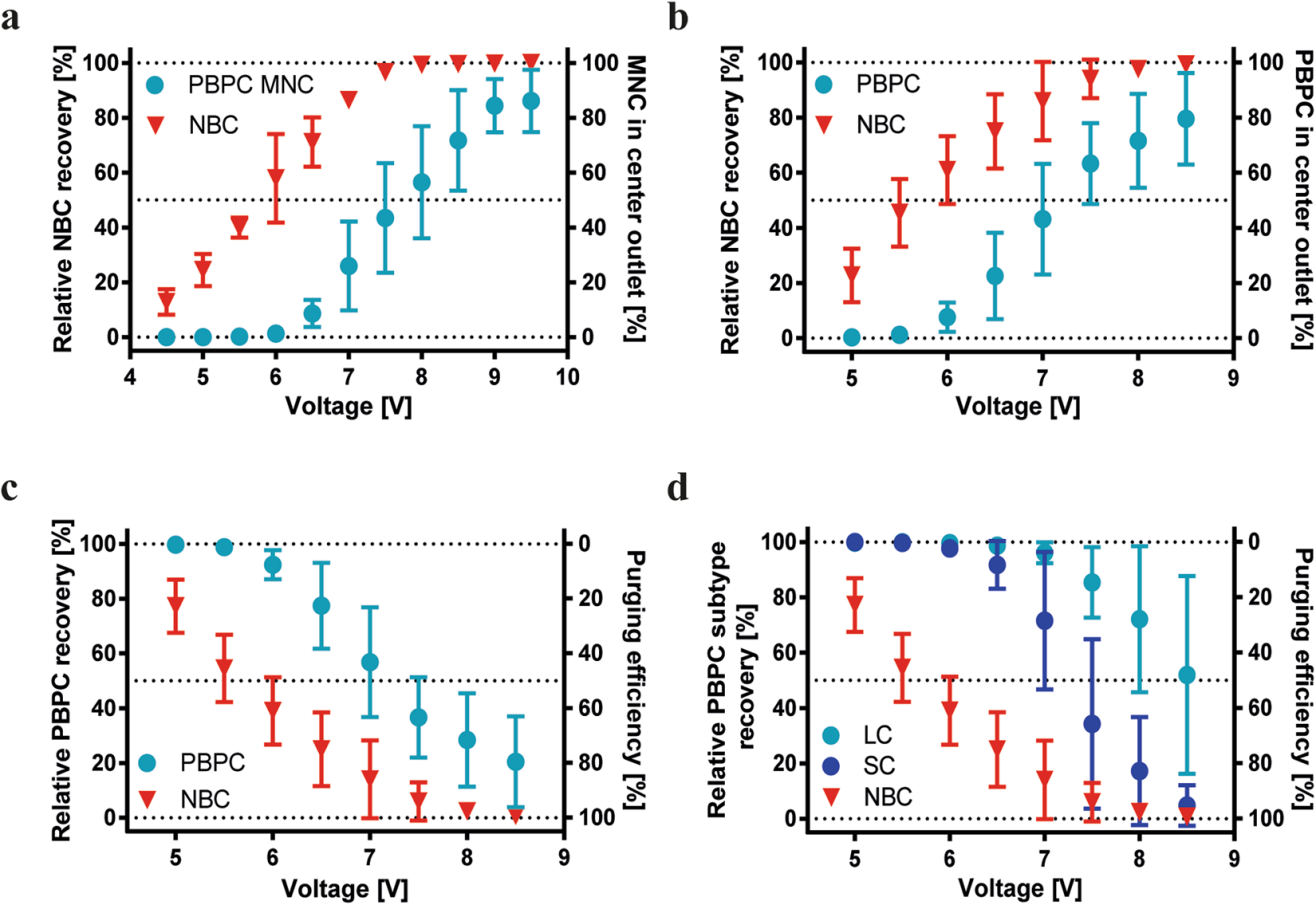
Acoustic separation of PBPC MNCs, unprocessed PBPCs and NBCs. Relative NBC recovery rates and fraction of (**a**) PBPC MNCs and (**b**) PBPCs collected in the center outlet for NBC enrichment experiments. (**c**) Relative PBPC recovery and NBC purging efficiency assessed in the side outlet in purging experiments. (**d**) Relative PBPC subtype recovery [%] of stem/progenitor cells (SC) and lymphocytes (LC) to NBCs and purging efficiency [%] (see equations 1, 2 in supplementary information). The samples were infused at 100 µl/min, which equals a throughput of 100,000 cells/min. Increasing voltages were used to actuate the separation channel transducer. Values are given as mean ± SD (n = 3–5).

First, NBC enrichment from PBPC MNCs was investigated (Fig. 4a) using a cell concentration of 1 × 10^6^ cells/mL and a spiking ratio of 1:100. For low voltages (here generated with 4.5–6.5 V) 91.36–99.99% of the PBPC MNCs were collected in the side outlet, while 12.84–71.16% of NBC were recovered in the center outlet with up to 89.62% purity (24.47% relative NBC recovery). As seen for blood MNCs, a pure population of NBCs could be enriched when accepting a lower recovery. On the other hand, higher recoveries could be realized when lower purities were accepted.

Next, NBC-spiked unprocessed apheresis samples were used to investigate if effective tumour cell enrichment and purging is possible from these more complex cell preparations. When aiming for NBC enrichment a maximum NBC purity of only 24.91% was achieved in the center outlet, while recovering 22.76% of the NBCs for the lowest voltage tested (5 V). This low purity was due to contaminating PBPCs in the center outlet and a low initial NBC concentration (Fig. 4b). A higher NBC recovery of 60.96% in the center outlet was achieved when increasing the voltage to 6 V, while only 7.65 ± 5.38% of the PBPCs were collected into this outlet.

When tumour cell purging from the graft is the aim, not only purging efficiency, but also a high PBPC recovery in the side outlet is a desired outcome (Fig. 4c). For low voltages (5–6 V) more than 90% of the PBPC were collected in the side outlet, while removing up to 60% of the tumour cells at 6 V. With increasing acoustic force more cells were directed to the center fraction and resulted in removal of NBCs from the side fraction by more than 1.5–2.3 log, but also an increased loss of PBPCs to the center fraction occurred. Figure 4d shows the relative recovery of lymphocytes and stem and progenitor cells, as well as NBCs in unprocessed PBPC samples (n = 3–5). The data showed a better discrimination of these PBPC subsets from NBCs as compared to the whole PBPC fraction (Fig. 4c). With increasing acoustic force, lymphocyte percentages collected in the side outlet ranged from 99.88 ± 0.11% to 51.93 ± 35.84% and relative recoveries of CD34+ cells ranged from 99.98 ± 0.04% to 4.78 ± 7.33%, indicating that these PBPC subsets displayed a higher acoustophoretic mobility. For the different voltages tested, CD34+ cells also showed a higher mobility than the smaller lymphocytes. Lymphocytes, NK, T helper, B and cytotoxic T cells showed lower acoustic mobilities than NBCs, while granulocytes and monocytes were more affected by the acoustic field (see Supplemental Fig. S4) and consequently moved to the center outlet prior to the other blood cell subtypes.

These results indicate that under the tested conditions, MNC losses occurred when aiming for high NBC depletion, i.e. effective purging. However, proof-of-principle was validated that tumour cell enrichment from PBPCs (MNCs) and purging of NBCs from PBPC products is possible. Best conditions for diagnostic NBC isolation were identified in the lower voltage range, at which 40–70% of the tumour cells were recovered in the center fraction and over 90% of the PBPCs were collected in the side outlet (here generated with 5–6 V). The most effective purging was achieved around 6.5–7 V, when more than 70% of the NBCs were collected in the center outlet and more than 70% of the CD34+ cells could be recovered in the side outlet.

### Acoustic separation did not affect cell proliferation capacity

**Figure 5:**
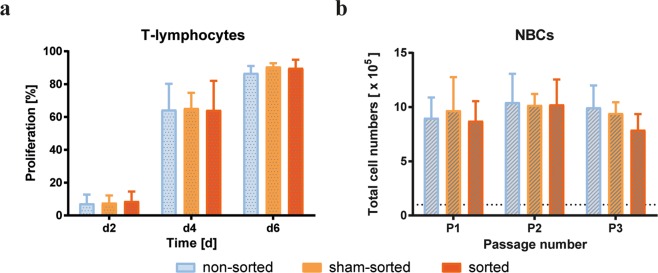
Analysis of T-lymphocyte and tumour cell proliferation capacity. (**a**) Non-sorted, sham-sorted and acoustically sorted MNC were stimulated with anti-CD3/CD28 and lymphocyte proliferation was measured based on CFSE staining intensities after 2, 4 and 6 days. Data are presented as percentage of dividing cells (n = 3). (**b**) Total number of cells of non-sorted, sham-sorted and acoustically sorted NBC after cultivating 100,000 initially seeded cells (dashed line) for 7 days over 3 passages. Two-way ANOVA analysis demonstrated no significant differences between the different groups in T-cell and NBC proliferation.

The proliferation capacities of T-lymphocytes and NBCs were investigated to determine a possible effect of the acoustic separation procedure on cell function (Fig. 5). The mean (±SD) percentage of proliferating non-sorted, sham-sorted and acoustically sorted T-lymphocytes was 7.3 ± 5.4%, 7.7 ± 4.6% and 8.7 ± 5.9% on day 2, 64.4 ± 15.8%, 65.3 ± 9.4%, and 64.3 ± 17.7% on day 4, and 86.7 ± 4.3%, 90.7 ± 2.0% and 89.8 ± 5.0% on day 6, respectively (Fig. 5a). The effect of acoustic separation on long-term NBC proliferation capacity was determined over 3 passages (Fig. 5b). Total cell numbers [×10^5^] after passage 1 (P1) were 9.0 ± 1.9 for non-sorted, 9.7 ± 3.1 for sham-sorted, and 8.7 ± 1.8 for acoustically sorted cells, 10.4 ± 2.6, 10.2 ± 1.0, and 10.2 ± 2.3 after P2, and 10.0 ± 2.0, 9.4 ± 1.0, and 7.9 ± 1.5 after P3, respectively. These results showed that there were no significant differences in proliferation of T-lymphocytes or NBC between acoustically sorted samples and controls.

## Discussion

Acoustophoresis is a gentle, non-contact, continuous, and label-free separation method for cells and particles. The ability to separate cells with acoustophoresis depends primarily on differences in the biophysical characteristics of cells/particles^9^. Highly discriminative label-free acoustophoretic separation has been demonstrated previously in various clinically relevant contexts, e.g. the separation of prostate and breast cancer cells from white blood cells, as well as the removal of platelets from PBPC products^5,11^. The current study served as the first step to establish acoustophoresis-based label-free neuroblastoma cell separation from peripheral blood and mobilized PBPC preparations, which are more complex biological samples containing a large number of different white blood cell types as well as haematopoietic stem and progenitor cells but which are depleted of red blood cells^11^. NBCs are considerably larger compared to platelets and generally smaller than most previously studied tumour cell types in acoustophoresis and other size-based separation methods^27^, and consequently selective tumour cell separation is considerably more challenging. Nevertheless, acoustic mobility measurements for PBPCs and NBCs using the method described by Cushing *et al*.^28^, which considers not only size but also acoustic contrast factor and medium viscosity, indicated that the mobility of NBCs was two times higher compared to PBPCs (data not shown). Accordingly, NBC enrichment from blood MNCs and PBPC products was realized in this work, and acoustophoresis could be evaluated as a potential purging technique for PBPC products.

Circulating tumour cells have been detected in blood and PBPC samples from neuroblastoma patients^15,29^. Diagnostic CTC collection, which is of high value for disease status determination and treatment evaluation, aims at high tumour cell recoveries and high purities sufficient for further downstream analysis. In contrast, PBPC purging aims at the maximum removal of tumour cells with minimum loss of stem/progenitor cells, whereas a loss of other non-transplant-relevant cells is acceptable. Currently applied PBPC purging methods, e.g. MACS or FACS, require antibody labelling and can be expensive, time and labour intensive. The development of alternative label-free approaches that allow for easy and effective tumour cell removal, for example realized as continuous in-line separation modules that can be added to existing apheresis equipment, is therefore justified.

For CTC enrichment and detection in blood, numerous methods and devices have been developed^30,31^, including spiral microfluidics^32^, microvortices^33,34^, magnetopheresis^35^, and dielectrophoresis^36^. Many CTC detection and enrichment methods rely on labelling of the target cells with a specific surface marker. These methods provide high sensitivity but are also highly dependent on the sufficient expression of a specific surface marker on the CTCs. Label-free methods, e.g. spiral and microvortice separation, show promising CTC separation, but have not yet shown their potential in processing and purging of complex PBPC products.

Acoustophoretic cell separation is largely based on cell size differences. Cell diameters of 6–10 µm have been reported for leukocytes, with lymphocytes ranging from 6.8–7.3 µm, monocytes from 9.0–9.5 µm, and granulocytes from 7.7–9.0 µm^37^. We confirmed size differences between the used neuroblastoma cells (10–20 µm) and peripheral blood cell preparations by Coulter Counter measurements (Fig. 2a), and accordingly, distinct acoustic mobilities were observed when tumour cells and blood cells were studied separately (Fig. 2b,c). The different cell types behaved similar but not identical in spiked samples which is due to the fact that increased complexities of mixed cell suspensions and an increased volume fraction of cells in the separation channel can lead to cell-cell interactions and cause carry-over between the outlets. Consequently, sample concentration is a critical parameter to obtain a highly resolved separation. In previous studies ~3 × 10^6^ cells/mL was proposed as a critical cell concentration for acoustophoretic separation in a comparable experimental set up, which is in agreement with our observations^5,6,38^ (Fig. 3a).

Our results showed that it is possible to isolate enriched NBCs from artificially tumour cell-contaminated mononuclear blood preparations, which might be sufficient for diagnostic downstream analysis using highly specific and sensitive detection methods such as quantitative RT-PCR^15^. Furthermore, our data demonstrate that adjustment of the acoustic field strengths by applying different voltages can be used to tune the system towards the requirements of different downstream analysis methods (Fig. 3c). For example, the system can be optimized for maximum purity while accepting lower recoveries, which is relevant for detection methods that are confounded by contaminating cells. In contrast, for other applications it might be of high importance to collect a larger number of NBCs while lower purities are not critical, e.g. when aiming to characterize tumour cell heterogeneity by single cell technologies.

The minimum tumour cell contamination concentrations tested so far equalled about one to five thousand NBCs per mL peripheral blood, and thus were about one hundred, three and ten times higher compared to numbers reported for peripheral blood, PBPC MNC preparations and unprocessed PBPCs^11,39^. In comparison, the FDA-approved immuno-labelling based CellSearch system has a detection limit of 5 CTCs per 7.5 mL blood^40^ and other microfluidic CTC enrichment approaches have reported detection limits as low as 1.25 CTCs/mL blood^41^. However, these microfluidic data were generated with tumour cells that were considerably larger than the NBCs used herein, making them difficult to compare with our results^27,33,34^. Nevertheless, tumour cell concentrations in our study were high and future optimization studies will therefore focus on the processing of lower and clinically more relevant tumour cell contamination levels. It should be noted that detection limits of 10 tumour cells/mL were recently realized with acoustophoretic separation in a similar experimental setup on lysed and fixed blood samples, demonstrating unperturbed separation performance of acoustophoresis at low tumour cell levels and hence indicating the feasibility of the technology for tumour cell separation at clinically relevant concentrations^5^.

Blood cell types like monocytes and granulocytes showed higher mobilities in the acoustic field (Supplemental Fig. S4a). NK cells, B cells, T helper cells and cytotoxic T cells on the other hand were collected in the side fraction (Supplemental Fig. S4b), confirming that similar sized cells showed comparable acoustic mobilities. In contrast to blood MNCs, a larger size overlap with NBCs was observed for all PBPC samples, which was also depending on the donor’s health status (Fig. 2a). Furthermore, increasing size distribution complexities were recorded, which was highest in unprocessed PBPCs (Fig. 2a). Thus, we used PBPC MNCs as an intermediate material between the less complex blood MNCs and complex unselected PBPCs for establishing acoustic PBPC processing.

Relative recoveries of NBCs from PBPC MNC samples were lower compared to blood MNCs, however, NBC enrichment could still be realized (Fig. 4a), even when processing unprocessed PBPC samples (Fig. 4b). Acoustophoretic NBC purging from PBPC samples showed a sufficient discrimination of PBPCs and NBCs that allowed for tumour cell depletion (Fig. 4b,c). Graft purging in autologous transplantation aims to remove contaminating tumour cells while simultaneously recovering stem and progenitor cells. Although the benefit of purging for high-risk neuroblastoma patients remains controversial^15,17,25,26^, we believe that every tumour cell removed from the graft will reduce the relapse risk, which justifies the further development of novel purging technologies. In the end, however, only larger studies with uniformly treated patient cohorts using effective purging, and collection of bigger sets of relevant data can finally provide an answer to this question.

In our experimental setup the most promising results for diagnostic NBC isolation achieved showed a 40–70% recovery of the tumour cells in the center outlet and >90% PBPC recovery in the side outlet. When aiming at purging, >70% of CD34+ cell recovery in the side outlet and more than 70% NBC depletion could be realized, showing the need for further optimization of the separation to be relevant for clinical application.

Widely used methods for PBPC purging in neuroblastoma are positive selection of CD34+ and tumour cell removal using immunomagnetic beads^15,17,23,42–46^. Donovan *et al*. reported >2 log depletion of NBCs (cell lines) from PBPC collections by magnetic CD34+ selection, and >2.4 to >4.6 logs of NBC depletion in clinical samples. Common labelling procedures are often cost and labour intense and require a suitable selection marker^42^. We depleted NBCs from PBPCs by 1.5-2.3 log using label-free acoustophoresis, which is in the lower range of what has been reported for other methods. Stem and progenitor cells (CD34+) show a higher acoustic mobility than the entire PBPC population but could still be discriminated from NBCs (Fig. 4d). However, CD34+ losses in the current experimental setup are too high and acoustophoretic properties of CD34+ cells need to be investigated in more detail to improve recovery in the tumour cell depletion setting. Due to low numbers of CD34+ cells (1.5–3.4%^11^) and especially low NBC numbers in the transplant it is furthermore necessary to apply sensitive tumour cell analysis methods such as quantitative RT-PCR to analyse clinically relevant samples, which will be addressed in future studies.

No negative impact of acoustophoresis on cell viability and function, including colony forming ability of acoustically sorted CD34+ cells, which is especially of high importance for transplantation, has been reported so far^11,12,47^. In this study, we confirmed that the proliferation capacity of T-lymphocytes and tumour cells was not compromised by exposure to acoustic forces or the microfluidic separation procedure.

Currently, the system is operated with a throughput of 100 µL/min. An up-scaling by at least factor 10 is needed to realize in-line purging in a standard apheresis machine. This would enable to use acoustic separation directly after the PBPC enrichment step before cells are transferred at standard flow rates of about 1 ml/min into the collection bag for later use for transplantation. However, we are confident that upscaling is possible, for example by operating several microchips in parallel or by adjusting the chip design, and we will thus approach this task in coming optimization experiments also focussing on processing samples with clinically relevant tumour cell contaminating rates.

Taken together, we herein report two important steps towards the development of clinical tumour cell enrichment and purging. First, we address the acoustic selection of neuroblastoma cells as new tumour cell type without prior fixation, and second, we establish acoustic tumour cell sorting on a new cell product, i.e. “real-life” patient and donor PBPCs, which have not been used previously for tumour cell work. Based on these results we will in future optimization experiments develop our method further towards clinical applicability. Here, some of the next steps – in addition to technical improvements and upscaling – will be to use patient derived xenograft tumour cells (PDX), which maintain the patient’s tumour characteristics over an extended time *in vitro*, and to furthermore decrease tumour cell contamination rates as well as to apply highly sensitive molecular methods for residual tumour cell quantification.

## Conclusion

This study provides proof-of-principle evidence that label-free acoustophoresis can be used to separate neuroblastoma cells from complex biological samples, such as peripheral blood mononuclear cells and peripheral blood progenitor cell products. Cell viability and proliferation capacity were not compromised by acoustic separation. Thus, acoustophoresis is a potentially valuable technology for the future development of effective label-free neuroblastoma cell enrichment and purging from peripheral blood and stem cell preparations.

## Materials and Methods

### Neuroblastoma cell culture

The human neuroblastoma cell line SH-SY5Y (Leibniz Institute DSMZ, German Collection of Microorganisms and Cell Cultures, Brunswick, Germany) was cultured in Dulbecco’s Modified Eagle’s Medium (Biochrom AG, Berlin, Germany) supplemented with 15% fetal bovine serum (Life Technologies, Carlsbad, CA, USA), and 100 IU/mL penicillin and 100 µg/mL streptomycin (Biochrom). Cells were cultured at 37 °C in a humidified atmosphere at 5% CO_2_. Cells were passaged at 80% confluence using 0.05% trypsin/0.02% ethylenediaminetetraacetic acid (EDTA) solution.

### Blood and peripheral blood progenitor cell samples

Blood samples obtained from healthy volunteers were collected in vacutainer tubes (BD Biosciences, San Jose, CA, USA) containing EDTA as anti-coagulant. Blood mononuclear cells (MNCs) were isolated by density gradient centrifugation using Ficoll-Paque PREMIUM (1.078 g/mL, GE Healthcare Bio-Sciences AB, Uppsala, Sweden). PBPC samples from healthy donors (n = 3, mean age 28.3 years) and patients (mantle cell lymphoma, n = 1; multiple sclerosis, n = 1; myeloma, n = 7, mean age 55.5 years) were obtained from leukapheresis products collected after standard mobilization treatment from the Clinical Apheresis Unit, Department of Clinical Immunology and Transfusion Medicine, Lund, Sweden. Details of the collection procedure have been described previously^11^. Apheresis products are enriched in white blood cell populations including stem and progenitor cells and highly reduced in red blood cells numbers. PBPC samples were either used without prior processing or as mononuclear cell preparations isolated by density gradient centrifugation. The collection and use of blood and PBPC samples, after informed consent, was approved by the Regional Ethical Review Board at the University of Lund. All experiments were performed in accordance with relevant guidelines and regulations.

### Cell counts and cell size measurements

Cells were counted with a NucleoCounter® NC-250™ (ChemoMetec A/S, Allerod, Denmark) according to manufacturer’s instructions and cell diameters were determined with a Multisizer 3 COULTER COUNTER^®^ (Beckman Coulter, Brea, CA, USA).

### Preparation of cell samples for acoustic separation

Cell samples were adjusted to a cell concentration of 1 × 10^7^ cells/mL in sorting buffer (Dulbecco’s Phosphate Buffered Saline w/o Ca^2+^/Mg^2+^ [Biochrom] and 5% Anticoagulant Citrate Dextrose Solution A [Terumo BCT, Inc. Lakewood, Colorado, USA]). Prior to separation, cells were stained with directly fluorochrome-conjugated monoclonal antibodies (BD Bioscience, San Jose, CA, USA) for 15 min in the dark at room temperature. Blood MNC and PBPC samples were labelled with CD45-APC (clone HI30), and NBCs with CD56-PE (clone MY31). T-lymphocytes and hematopoietic stem and progenitor cells were stained with CD3-FITC (clone SK7) and CD34-PerCP-Cy5.5 (clone 8G12), respectively. Following staining, cell concentrations were adjusted to 1 × 10^6^ cells/mL if not otherwise indicated. Labelled tumour cells were spiked into the samples at ratios from 1:1 to 1:1,000 with 1:100 being used as the standard ratio in the majority of experiments.

### Acoustophoresis chip design and set-up

The acoustophoresis chip design (Fig. 1) and the fabrication process have been previously described in detail^6^. The chip has a sample inlet followed by a prealignment channel (20 mm × 300 μm × 150 μm), a v-shaped flow splitter with a central inlet for sorting buffer infusion, leading into the main separation channel (30 mm × 375 μm × 150 μm), ending in a trifurcation with one central outlet and two side branches ending in one common side outlet. The prealignment channel was actuated by a piezoceramic transducer (PZ26, Ferroperm Piezoceramics, Kvistgaard, Denmark), resonant at 5 MHz and the separation channel by a second transducer resonant at 2 MHz. The transducers were actuated with a dual channel function generator (AFG3022B, Tektronix, Beaverton, OR, USA), equipped with amplifiers (in-house build), and connected to a two-channel oscilloscope (TDS 2002C, Tektronix) for voltage and frequency monitoring. The temperature of the separation channel was measured and controlled by a PID control loop using a Peltier-controller (CoolTronic GmbH, Beinwil am See, Switzerland), a Peltier element (Farnell, London, UK) and a Pt1000 temperature detector (Farnell). A pressure driven setup controlled by three flow sensors (Liquid Flow Meter CMOSens® SLI-1,000, Sensirion AG, Staefa, Switzerland)^48^ and LabVIEW software (National Instruments Corporation, Austin, TX, USA) realized the continuous flow in the system. Cell-free sorting buffer solution was infused with a flow rate of 300 µL/min, outlet flow rates were 100 µL/min in the center outlet, and 300 µL/min in the side outlet, resulting in a sample inlet flow rate of 100 µL/min. The separation principles of the chip are illustrated in Fig. S1 and video animations S1 and S2.

### Acoustic separation procedure

Initial calibration of the acoustophoresis system to determine the optimum frequency was performed visually by using 5 µm and 7 µm polystyrene microspheres (Sigma-Aldrich, St.Louis, MO, USA) and validation of the bead separation performance by flow cytometry (FACS Canto II flow cytometer with FACSDiva software, BD Biosciences). The pre-stained cell sample was connected to the sample inlet and aspirated into the pre-focusing channel (Fig. 1). Two-dimensional prealignment was induced by operating the prealignment transducer at a frequency of 4.928 MHz and a voltage of 5 V. In the separation channel, cells migrated towards the pressure node in the channel center under the influence of the acoustic force generated by the separation transducer, operated at a frequency of 2.006 MHz. A range of different separation transducer voltages (4–10 V) was applied to investigate voltage effects on separation outcome.

### Analysis of sorted cell samples

Acoustically sorted cells collected in the center and side fractions were analysed by flow cytometry using a FACS Canto II flow cytometer with FACSDiva software (BD Biosciences). Propidium iodide (1 µg/mL, Sigma-Aldrich) or 7-aminoactinomycin D (200 µg/mL, Sigma-Aldrich) were used for dead cell exclusion. Total leukocytes were identified as CD45+, NBCs as CD45-/CD56+. T-lymphocytes were determined as CD45+/CD3+ leukocytes, and hematopoietic stem and progenitor cells as CD45+/CD34+. Data were analysed with FlowJo v10 software (FlowJo LLC, Ashland, OR, USA). Relative (subtype) recoveries/purging efficiencies and purities were calculated as follows:

1 $$ \% \,Relative\,recover{y}_{center}=\frac{{C}_{C}\times {V}_{C}}{{C}_{C}\times {V}_{C}+{C}_{S}\times {V}_{S}}\times 100 \% $$

2 $$ \% \,Relative\,recover{y}_{side}=\frac{{C}_{S}\times {V}_{S}}{{C}_{C}\times {V}_{C}+{C}_{S}\times {V}_{S}}\times 100\, \% \,$$

3 $$ \% \,Purit{y}_{outlet}=\frac{{C}_{outlet}\times 100\, \% \,}{{C}_{total}}$$

where C_c_ and C_s_ are the cell concentrations in center and side fraction, V_c_ and V_s_ are the volumes of the center and side fraction, C_outlet_ is the cell concentration in a given outlet and C_total_ is the total cell concentration in the two outlet fractions.

### T-Lymphocyte and tumour cell proliferation assays

The proliferation capacity of acoustically separated T-lymphocytes was evaluated by stimulating carboxyfluorescein diacetate succinimidyl ester (CFSE) stained cells with anti-CD3/anti-CD28 antibodies (clones HIT3a/CD28.2, eBiosciences), as described previously^47^. Briefly, MNCs were labelled with CFSE (5 µM, Invitrogen Life Technologies, Thermo Fisher Scientific, Waltham, MA, USA) and seeded in triplicates at 20,000 cells per well in 96-well plates (Falcon®, Fisher Scientific). Non-sorted cells and sham-sorted cells (ultrasound off) served as controls. CFSE fluorescence intensity was measured with flow cytometry every other day for six days (FACS Canto II with FACSDiva software and FlowJo software). T-lymphocytes were identified by anti-CD3-APC (clone HIT3a) co-staining.

Tumour cell proliferation was determined for sorted, non-sorted and sham-sorted tumour cells that were seeded at 100,000 cells per well in triplicates in 6-well plates (Falcon^®^) and cultivated for 7 days over 3 passages (P). Total and viable cell numbers were determined with a NucleoCounter NC-250 (ChemoMetec A/S).

### Statistical analysis

Results are presented as mean ± standard deviation (SD). Statistical significance was determined by two-way ANOVA for proliferation experiments (Prism 7.03 software package, GraphPad, San Diego, CA).

## Supplementary information

Supplementary Information

Video S1. Acoustophoresis chip overview

Video S2. Buffer inlet and center outlet areas

## Data Availability

All data that support the findings of this study are provided in the article and its Supplementary Information. Raw data are available on request.
