# Supplementary material for: Label-free neuroblastoma cell separation from hematopoietic progenitor cell products using acoustophoresis - towards cell processing of complex biological samples
Source: Sci Rep. 2019 Jun 19;9:8777. doi: 10.1038/s41598-019-45182-3 (PMC6584513; doi:10.1038/s41598-019-45182-3)
Supplement: Supplementary file 1 — Supplementary Information [file 41598_2019_45182_MOESM1_ESM.pdf]

Supplemental Information

Label-free neuroblastoma cell separation from hematopoietic progenitor cell products using acoustophoresis - towards cell processing of complex biological samples

Franziska Olm, Anke Urbansky, Josefina H. Dykes, Thomas Laurell, and Stefan Scheding

Equations

The primary axial radiation force (F_{Ax}, (1)) directs particles into acoustic pressure nodes or antinodes depending on their acoustic contrast factor Φ (2) and scales with the particles radius (r), the acoustic energy density (E), the wavenumber (k = 2π/λ), where λ is the wavelength, and x the particles position in the propagation direction of the wave. The contrast factor is dependent on differences in the density (ρ) and compressibility (κ) of the particles (p) and the surrounding medium (o).

$$\boldsymbol{F_{Ax}} = \boldsymbol{4\pi\, r^3 E\, k\, sin(2kx)}\, \Phi \qquad \textbf{(1)}$$

$$\Phi = \frac{\boldsymbol{\kappa_o - \kappa_p}}{\boldsymbol{3\kappa_o}} + \frac{\boldsymbol{\rho_p - \rho_o}}{\boldsymbol{2\,\rho_p + \rho_o}} \qquad \textbf{(2)}$$

Supplemental Figures

Figure S1

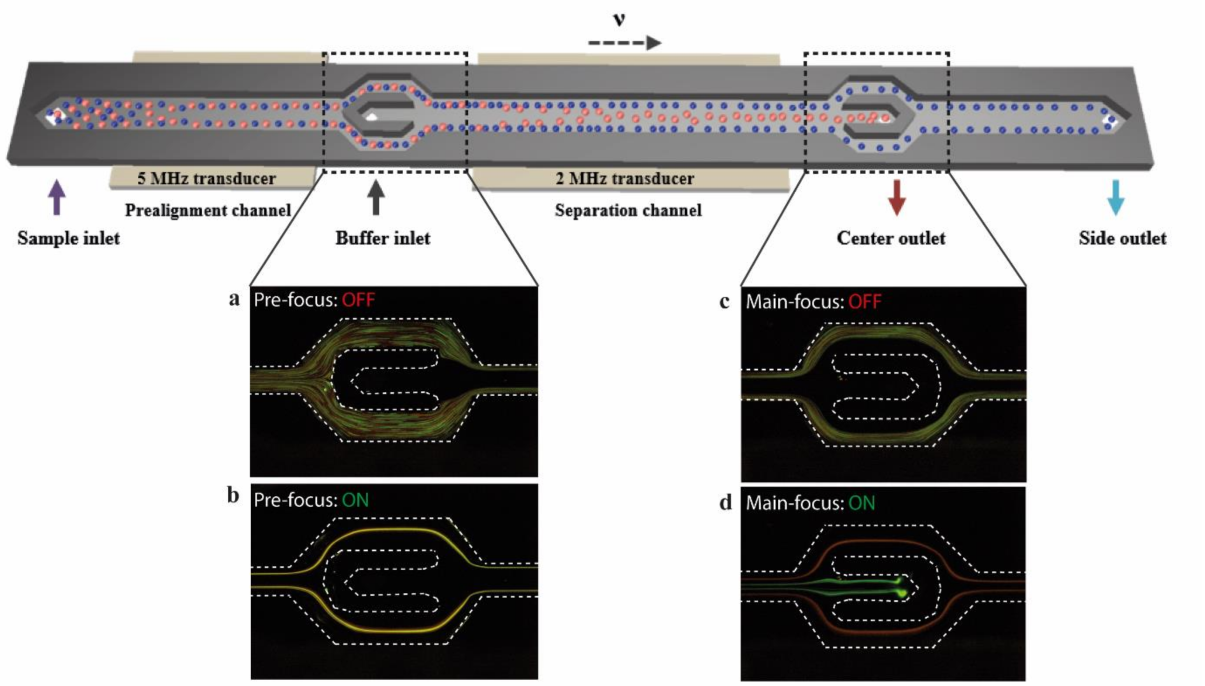

Figure S1. Visualization of the separation process in the acoustophoresis chip.

The schematic drawing shows an overview of the acoustic device as presented in Figure 1. For fluorescence images 4 µm Fluoro-Max green fluorescence polymer microspheres and 2 µm Fluoro-Max red fluorescence polymer microspheres (Thermo Fisher Scientific, Waltham, MA, USA) dispersed in MilliQ + 0.01% Triton-X-100 (Sigma-Aldrich, St. Louis, MO, USA) were used. Pictures S1a and b show the beads in the buffer inlet area. When the pre-focus is turned off (a), particles move randomly distributed with the laminar flow through the chip. When the pre-focus is turned on, the particles are aligned in the two pressure nodes in the prealignment channel by the acoustic forces and flow through the chip in a defined and ordered manner (b). With pre-focus (a) and main-focus off (c), all particles move close to the channel walls and no separation takes place, i.e all particles exit the chip trough the side outlet (c). With main-focus on (d) the aligned particles enter the separation channel and particles with higher acoustic mobility are collected in the center outlet (green fluorescent particles, red in schematic).

Smaller particles (red fluorescent particles, blue in schematic) are less affected by the acoustic field and stay closer to the channel walls (d). They are collected in the side outlet.

Figure S2

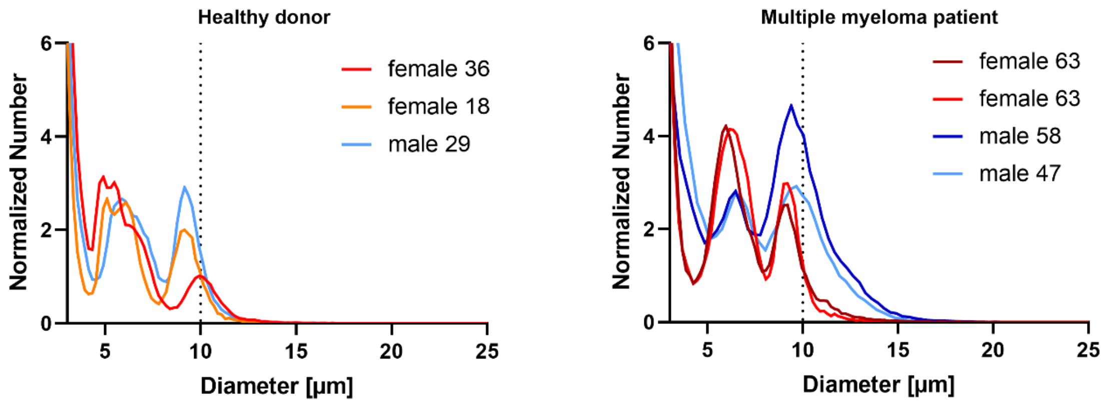

Figure S2: Cell size distributions of different peripheral blood progenitor cell products.

Samples were obtained from healthy donors (left) and multiple myeloma patients (right). Age and gender information are provided in the legends. Cell numbers are shown as normalized cell counts relative to the total number of cells acquired for each individual sample. Small sized counts account for dead or apoptotic cells as well as debris in the samples. Dotted vertical lines represent the lower size limit of NB tumour cells.

Figure S3

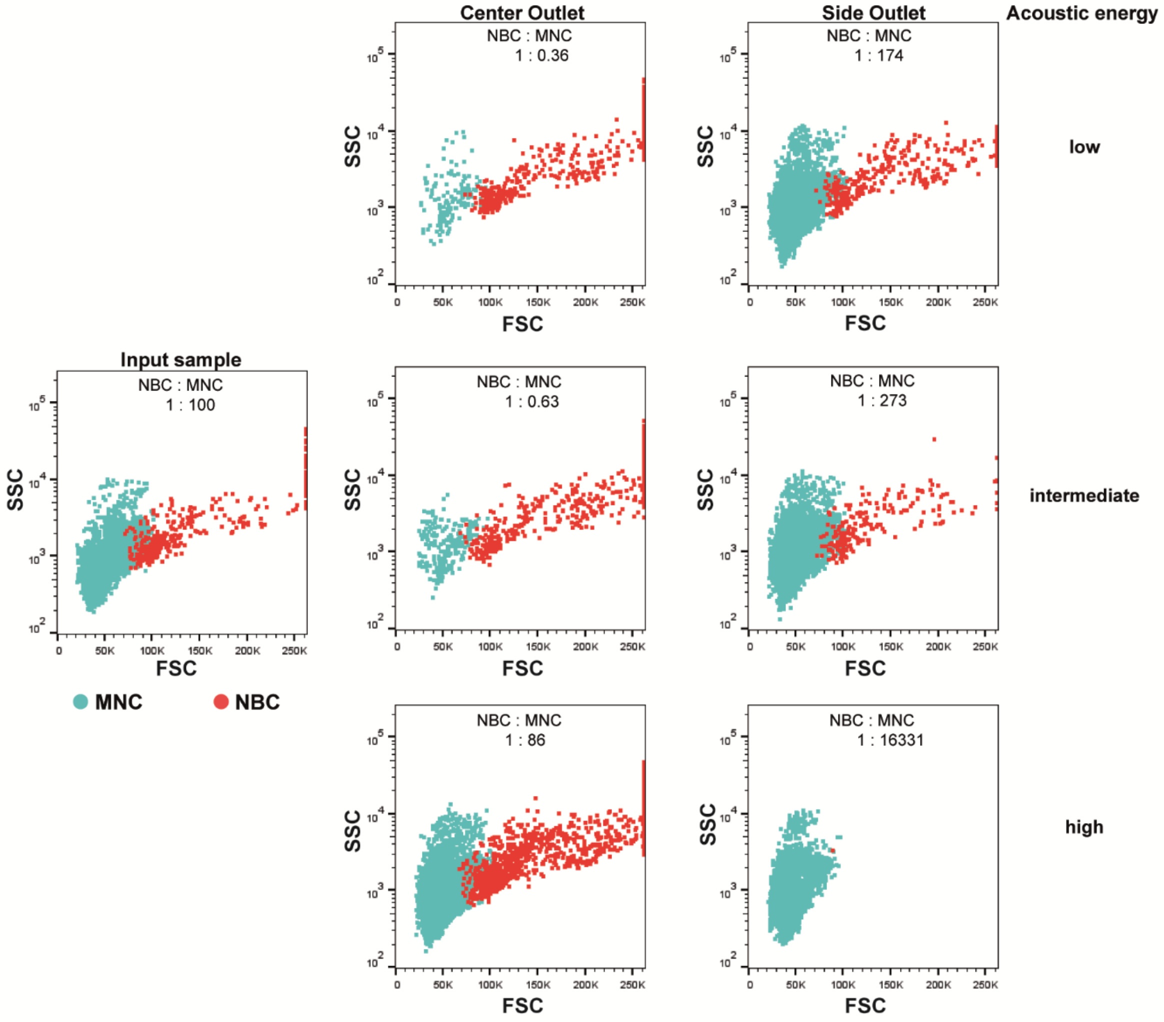

Figure S3 (related to Figure 3c). Acoustic separation of blood MNC and NBC

SSC/FSC flow cytometry plots of representative input (left), center (middle) outlet and side (right) outlet results for blood MNC (red) and NBC (blue) separation. MNCs and NBCs are identified based on expression of CD45 (MNC) and CD56 (NBCs). Samples were separated with low (upper row), intermediate (middle row), and high acoustic energy (lower row).

Figure S4

a

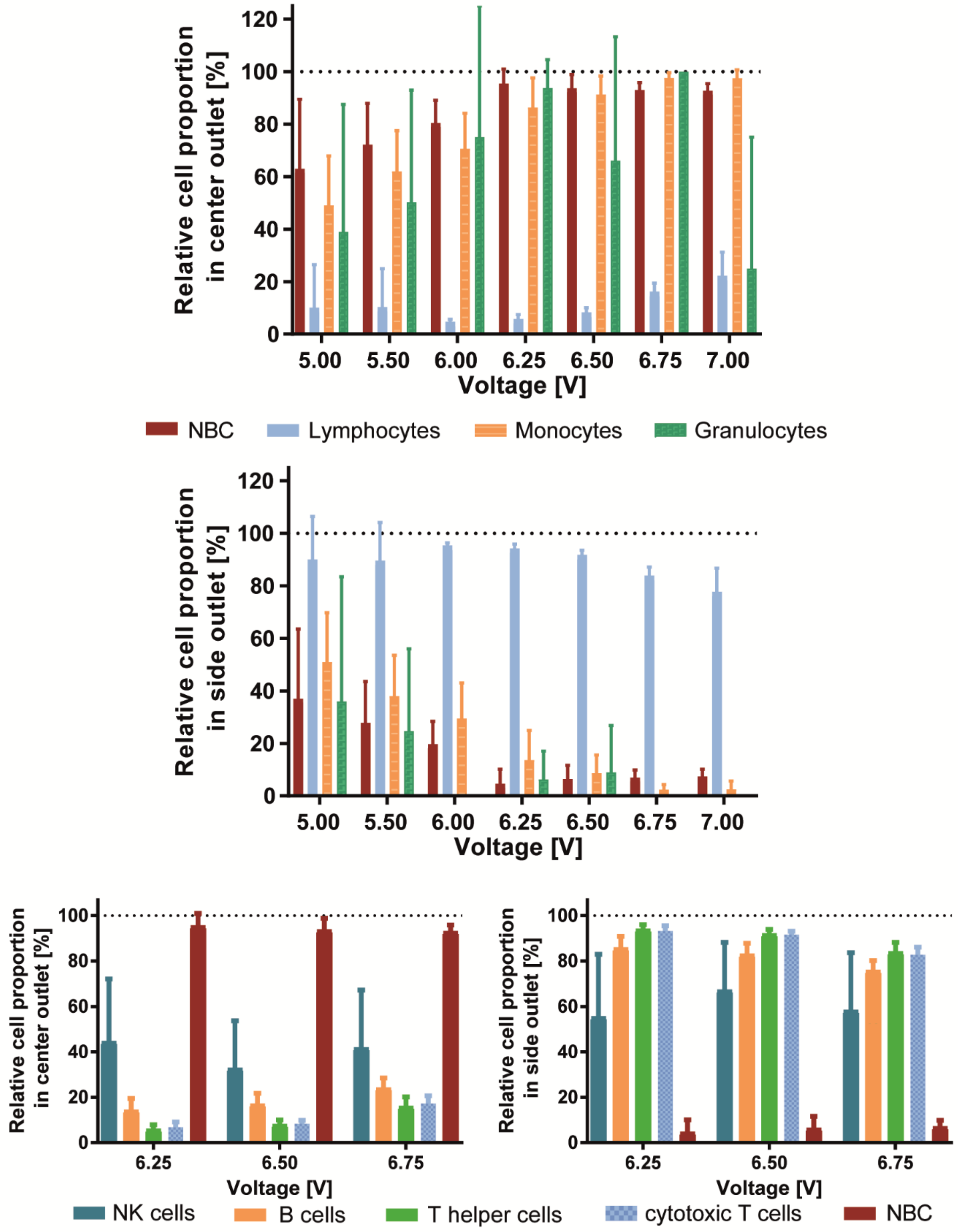

Figure S4 (related to Figure 3). Acoustic separation of blood MNC and NBC.

Distribution of different leukocyte subpopulations and tumour cells under different acoustic field forces. The relative fraction [%] of lymphocytes, granulocytes, monocytes and tumour

cells (a), and of NK cells, T helper cells, B cells and cytotoxic T cells (b) are shown for the center (a, upper, b left) and side (a lower, b right) fraction relative to all cells of a particular cell type in the two outlets. Data are presented as mean with SD (n=3).

Cells were stained with directly fluorochrome-conjugated monoclonal antibodies (BD Bioscience, San Jose, CA, USA) CD45 (clone HI30), CD56 (clone MY31), CD3 (clone SK7), CD14 (clone M5E2), CD66b (clone G10F5), CD19 (clone HIB19), and CD8 (SK1), respectively, for 15 min in the dark at room temperature and analysed by flow cytometry.

Supplemental Videos

For the videos 2 µm Fluoro-Max red fluorescence polymer microspheres and 4 µm Fluoro-Max green fluorescence polymer microspheres (Thermo Fisher Scientific) in MilliQ + 0.01% Triton-X-100 (Sigma-Aldrich) as buffer were used.

Video S1. Overview of the microfluidic chip with ultrasound turned off and on moving from buffer inlet area to the outlet area in flow direction. When the ultrasound is turned off, particles move randomly through the chip with the laminar flow and no separation or focusing takes place. When the ultrasound is turned on particles are focused and pre-aligned in the pre-focus area and separated in the separation channel. Green particles show higher acoustic mobility and are collected in the center outlet while red particles move slower in the acoustic field and stay closer to the channel walls and exit through the side outlet.

Video S2. Buffer inlet and center outlet areas visualizing the effects of turning pre-focus and main-focus on and off. First, the buffer inlet area after the pre-focusing channel is shown. When the pre-focus is turned off particles flow with the laminar flow and are distributed in the whole channel. Then the pre-focus is turned on and particles get focused and aligned, which is reversed when the pre-focus is turned off again. The particles continue to the center outlet area with the laminar flow and get aligned when the pre-focus is turned on but stay in the flow lines and consequently close to the channel walls. Only when the main-focus is turned on, the green particles are focused to the center outlet and separated from the red particles. When the pre-focus is turned off the 2D focusing effect is lost and the separation resolution decreases, which demonstrates the importance of the pre-focusing step for achieving high discrimination between the different sorted particles/cells.
